# Multicolour-banding fluorescence *in situ* hybridisation (mbanding-FISH) to identify recurrent chromosomal alterations in breast tumour cell lines

**DOI:** 10.1038/sj.bjc.6602228

**Published:** 2005-01-18

**Authors:** A Letessier, M-J Mozziconacci, A Murati, J Juriens, J Adélaïde, D Birnbaum, M Chaffanet

**Affiliations:** 1 Laboratory of Molecular Cytogenetics, Department of Molecular Oncology, Paoli-Calmettes Institute-UMR599 INSERM, Marseille Cancer Research Institute, Marseille, France; 2 Department of Biopathology, Paoli-Calmettes Institute, Marseille, France

**Keywords:** breast cancer, chromosome, fluorescence *in situ* hybridisation, translocation

## Abstract

Recurrent chromosome breakpoints in tumour cells may point to cancer genes, but not many have been molecularly characterised. We have used multicolour-banding fluorescence *in situ* hybridisation (mbanding-FISH) on breast tumour cell lines to identify regions of chromosome break created by inversions, duplications, insertions and translocations on chromosomes 1, 5, 8, 12 and 17. We delineate a total of 136 regions of break, some of them occurring with high frequency. We further describe two examples of dual-colour FISH characterisation of breakpoints, which target the 1p36 and 5p11–12 regions. Both breaks involve genes whose function is unknown to date. The mbanding-FISH strategy constitutes an efficient first step in the search for potential cancer genes.

Many loci that contribute to mammary oncogenesis remain probably to be discovered. Only a handful of genes have been demonstrated to have a direct role in mammary oncogenesis after alteration. At the chromosomal level, amplifications, translocations and deletions point to the existence of a potential cancer gene in an affected region. Genes such as *ERBB2* and *CCND1*, which encode a tyrosine kinase receptor and a G1 cyclin, respectively, are likely the selected oncogenes of the 17q12 and 11q13 amplification, respectively. For other amplification regions, such as the 8p12 and 20q13 regions, and for deletions, the identity of the cancer genes remains uncertain. The characterisation of translocations has provided additional cancer gene candidates, such as *FHIT* (Popovici *et al*, 2002), *NTRK3* and *ETV6* (Tognon *et al*, 2002; our unpublished observations), *BCAS* (Bärlund *et al*, 2002) and *NRG1* (Wang *et al*, 1999; Adélaïde *et al*, 2003; Huang *et al*, 2004) genes. Given the number of recurrent chromosomal breaks observed in breast cancer (http://cgap.nci.nih.gov/Chromo somes/RecurrentAberrations), this line of research might become more fruitful than the search for deletions through loss of heterozygosity data, which has been rather disappointing (Devilee *et al*, 2001). Although the mechanisms and consequences of translocations are not as clear as for those found in hemopathies and sarcomas, the existence of recurrent events suggests a role for the genes located at the breakpoints and provides a relatively easy way to discover more cancer genes.

A major limitation of this approach, however, is the difficulty to obtain reliable informative data on chromosomes of breast tumours. Technologies such as chromosome painting, spectral karyotyping (SKY) and multiplex- or multicolour-fluorescence *in situ* hybridisation (M-FISH) (Schröck *et al*, 1996; Speicher *et al*, 1996) can overcome the limitations of conventional cytogenetical methods in the characterisation of complex chromosome alterations observed in cancers. They allow the simultaneous visualisation of all human chromosomes in different pseudocolours and a better characterisation of the rearrangements. However, intrachromosomal rearrangements or the origin of abnormal chromosomal segments cannot be defined with these techniques. To overcome these limitations, different multicolour-banding techniques including mbanding-FISH have been developed (Müller *et al*, 1997, 1998; Chudoba *et al*, 1999; Liehr *et al*, 2002a, 2002b; Kakazu *et al*, 2003). They can be performed with combinations of well-defined subregional probes (Müller *et al*, 2004). They can reveal aberrations not visible by other methods, and consequently facilitate the molecular identification of targeted genes. Obtaining good metaphase cells in breast tumours is still difficult but cell lines may, in a first step, be used to evaluate the usefulness of the approach.

We have used mbanding-FISH to search for regions of recurrent alterations in breast tumour cell lines on five chromosomes. We have identified several such regions. We show that mbanding-FISH is a powerful method to identify rapidly regions of chromosomal breaks. We report two examples of further characterisation of breakpoints, which target the 1p36 and 5p11–12 regions. This technology should constitute an efficient first step in the search for potential cancer genes.

## MATERIAL AND METHODS

### Tumour cell lines

In all, 20 established breast tumour cell lines were used. They were as follows: Br-Ca-MZ-01 (Möbus *et al*, 1998), BT-474, CAMA-1, HCC1937 (Tomlinson *et al*, 1998), MCF-7, MCF-10F, MDA-MB-157, MDA-MB-175, MDA-MB-231, MDA-MB-361, MDA-MB-453, MDA-MB-468, SK-BR-3, UACC-812, ZR-75-1 (ATCC, Rockville, MD, USA), SUM-44, SUM-52, SUM-149, SUM-185 (Forozan *et al*, 1999; http://www.cancer.med.umich.ed u/breast_cell/clines/clines.ht ml) and IPC-BC-116. We established the latter cell line from an inflammatory carcinoma, after informed consent of the patient; the tumoral karyotype was established and displayed a t(1;6)(p36;p21) translocation as sole abnormality. All lines are derived from carcinomas except MCF-10F, which is derived from a fibroadenoma. The cells were grown using the recommended culture conditions, while IPC-BC-116 was grown in DMEM/Ham F12 medium (1/1) supplemented with 10% FBS, 10 *μ*g ml^−1^ Insulin, 1 *μ*g ml^−1^ hydrocortisone and 10 ng ml^−1^ EGF.

### Mbanding-FISH

The harvesting of cells, fixation and preparation of metaphase spreads for chromosome banding analysis and FISH were carried out from cytogenetic pellets of cultured cell lines according to protocols described in Courtay-Cahen *et al* (2000). Mbanding-FISH, a high-resolution multicolour-banding technique that provides precise information on intrachromosomal rearrangements and exact breakpoint mapping, was performed on chromosome metaphases cell lines (Table 1) with probe chromosome 1-, 5-, 8-, 12- and 17-mband cocktails, according to the protocol recommended by Metasystems (Chudoba *et al*, 1999) and as described previously (Popovici *et al*, 2002). The commercial probe mband cocktail derives from microdissected region-specific partial chromosome paints (PCP). Each PCP is labelled using a unique fluorochrome combination and partially overlaps with the neighbouring one.

The specific chromosome-mband cocktail was denatured and hybridised on treated and denatured metaphase chromosomes, according to the manufacturer's protocol. After hybridisation, chromosomes were washed with 50% formamide/2 × SSC and 2 × SSC at 42°C. The excitation/emission spectra of the fluorochromes are equivalent to FITC, Spectrum Orange™, TexasRed® and DEAC (diethylamino-coumarin (www.metasystems.de)). For chromosome 1-, 5-, 8- and 12-mband cocktail probes, an additional labelling was carried out using biotin, which is detected by Streptavidin-Cy™5 (B-tect).

After counterstaining with 4,6-diamidino-2-phenylindole, the images were analysed with a microscope (DMRXA, Leica Microsystèmes, Marseille, France), captured with a CCD camera, filtered and processed with ISIS software (*In Situ* Imaging Systems, Metasystems Hard- und Software GmbH, Altlussheim, Germany) (described in www.metasystems.de). The resulting fluorescence intensity pattern along the chromosome axis shows a continuous change of colour ratios. Pseudocolours can be assigned to chromosome sections of similar colour ratios giving rise to a reproducible banding pattern that does not depend on chromosome condensation (Chudoba *et al*, 1999).

A region of breakpoints was defined by comparison between the abnormal colour spectrum of a derivative chromosome and the normal profile of the corresponding nonaltered chromosome; a disruption of colour-banding pattern defines a region of break. Amplifications by duplication or triplication were defined on the basis of duplicate or triplicate of at least two colour sections observed on the abnormal colour spectrum and pseudocolour profile of a derivative chromosome. At least 10 metaphases exhibiting the same derivative chromosomes were studied, and mbanding colour profiles were analysed for each derivative.

### FISH analysis

To delineate the t(1;6)(p36;p21) and t(5;12)(p12;p11) events, and identify the potential genes involved, dual colour FISH analysis was carried out using labelled DNA of BAC clones as probes as described previously (Adélaïde *et al*, 2003) on IPC-BC-116 and UACC-812 breast tumour cell lines, respectively.

To refine rapidly the 1p36 breakpoint in the t(1;6)(p36;p21) rearrangement, the following BAC clone set covering this region was selected with variable genomic distance between clones: tel-RP11-164A22 (AC055792; chr1:14,996,330-15,182,825), RP11-430L17 (AL358794; chr1:16,386,491-16,511,439), RP11-473A10 (AL358593; chr1:17,656,749-17,799,202), RP11-99F3 (AC020587; chr1:18,111,224-18,284,657), RP11-294O9 (AC026576; chr1:19,390,941-19,574,586), RP11-97J18 (AL391598; chr1:19,804,646-19,980,341), RP11-200J11 (AC022786; chr1:20,673,314-20,851,807) and RP11-487E1 (AL627311; chr1:21,041,056-21,210,231)-cen. To refine the 5p12 breakpoint, we selected BAC clones included in the region, close to, or contained in the 876F7 YAC clone previously described spanning the breakpoint at 5p12 (Popovici *et al*, 2002): tel-RP11-453A8 (AC027488; chr5:40,533,761-40,705,000), RP11-30F7 (AC016332; chr5:41,237,134-41,386,355), RP11-9G14 (included in NT_006576 and mapped by FHCRC lab; chr5:41,319,672-41,319,970), RP11-190J8 (AC021600; chr5:41,384,203-41,536,708), RP11-184C11 (AC025649; chr5:41,472,703-41,639,760)-cen. DNA from BAC clones were purified, labelled and used as probes in combination with centromeric probes specific for chromosome 6 (revealed in green, FITC) or digoxigenin-labelled centromeric probe specific for chromosome 12 (revealed in red, TRITC). All BAC clones were obtained from the BACPAC resource (Children's Hospital Oakland - BACPAC Resources, Oakland, CA, USA). Image analysis was carried out as described in the previous section.

## RESULTS

### Regional localisation of chromosomal breakpoints on chromosomes 1, 5, 8, 12 and 17 in breast tumour cell lines

**Figure 1:**
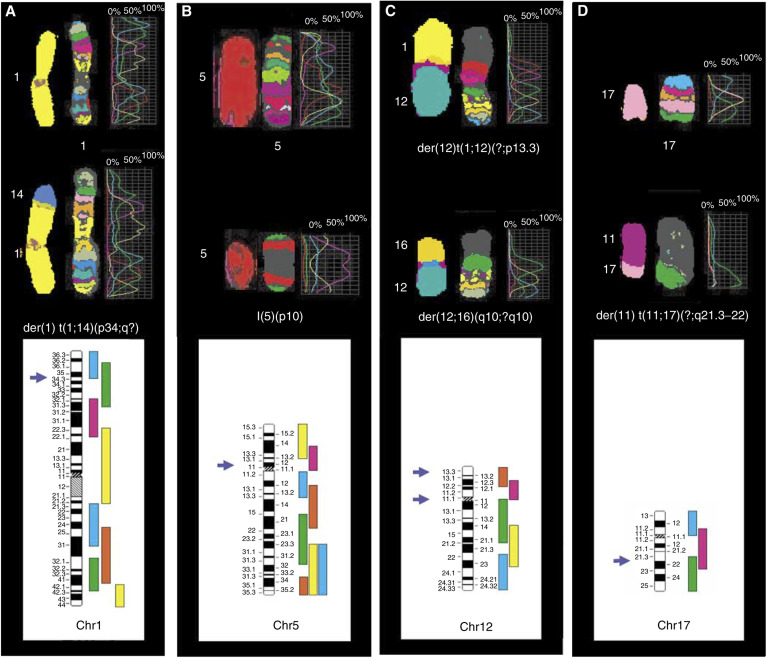
Examples of mbanding-FISH in breast tumour cell lines. MDA-MB-231 (**A**), MDA-MB-157 (**B** and **C**) and ZR-75-1 (**D**) were analysed by FISH with specific mband cocktail probes for chromosome 1 (**A**), 5 (**B**), 12 (**C**) and 17 (**D**), respectively. M-FISH images of normal (save for chromosome 12 in MDA-MB-157 (**C**)), and derivative chromosomes 1, 5, 12 and 17 previously characterised in these cell lines (Popovici *et al*, 2002) are shown on the left hand of the corresponding mband images (pseudocolour profile) defined by colour spectra (on the right). Regional locations of breakpoints targeting der(1)t(1;14)(p34;q?) (**A**), I(5)(p10) (**B**), der(12)t(1;12)(?;p13.3) (**C** upper part), der(12;16)(q10;?q10) (**C** middle part) and der(11)(t(11;17)(?;q21.3–22) (**D**) present in MDA-MB-231 (**A**), MDA-MB-157 (**B** and **C**) and ZR-75-1 (**D**), respectively, were assigned by comparison with the normal profile. They are indicated by arrowheads on the corresponding ideograms (bottom part), which exhibit on their right hand the sequence of microdissected region-specific PCP labelled using a unique fluorochrome combination defining then the normal colour spectrum. Each PCP was labelled and partly overlaps with the neighbouring one.

We chose to study five chromosomes by mbanding-FISH, that is, 1, 5, 8, 12 and 17. The reasons of our choice were the following: these chromosomes have different sizes and are affected with various frequencies in breast cancer (Kytölä *et al*, 2000; Teixeira *et al*, 2002) (Table 1). Examples of mbanding-FISH are illustrated in Figure 1 showing breakpoints on der(1)t(1;14)(p34;q?), i5(p), der(12)t(1;12)(?;p13.3), der(12;16)(q10;?q10) and der(11)(t(11;17)(?;q21.3–22) derivative chromosomes observed in MDA-MB-231 (Figure 1A), MDA-MB-157 (Figures 1B and 1C) and ZR-75-1 (Figure 1D), respectively. A region of breakpoint was defined by comparison between the abnormal colour spectrum of a derivative chromosome and the normal profile of the corresponding nonaltered chromosome. The regions are indicated on the corresponding ideograms (Figures 1A–D, bottom part). For each of the cell line studied, one to several derivative chromosomes could be observed. Comprehensive results in agreement to ISCN (1995) are described as supplementary data (Supplementary Table 1). For economical reasons, when previous experiments (e.g. with M-FISH) had demonstrated integrity of one or more of the five selected chromosomes in a cell line, this cell line was not studied with the respective mbanding probe. This explains why not all cell lines were systematically investigated with the five chromosome probes.

**Figure 2:**
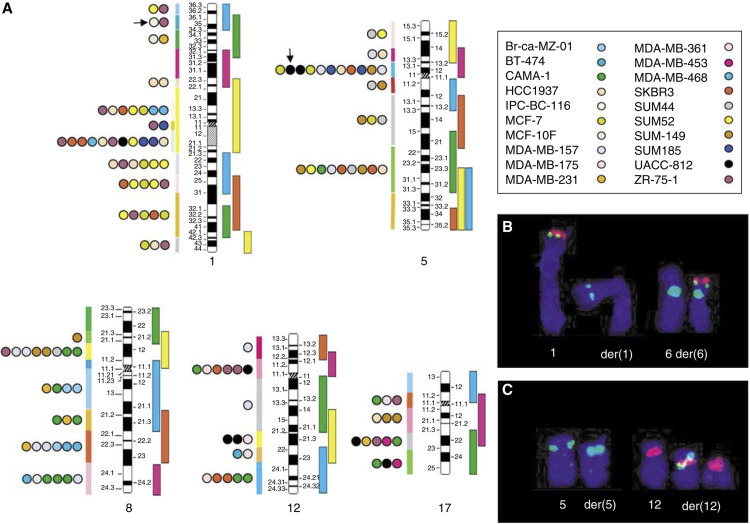
(**A**) Regional distribution of breakpoints in breast tumour cell lines. From mbanding-FISH analysis on breast tumour cell lines, a total of 136 breakpoints were described (Supplementary Table 1). Each coloured circle corresponds to a cell line as defined in the inset in upper right corner. These circles are positioned on ideograms of normal 1, 5, 8, 12 and 17 chromosomes in the region affected by breakpoints characterised in cell lines derivatives reported in the supplementary table. Each coloured circle represents a breakpoint observed in one derivative. The occurrence order of coloured circles follows the order of breakpoints characterised in the corresponding cell lines reported in the supplementary table, for example, two circles with the same colour on the same region mean that this region is involved in two different derivatives present in the same cell line. Black arrows show 1p36 and 5p12 breakpoints of the t(1;6)(p36;p21) and t(5;12)(p12;p11) present in IPC-BC-116 and UACC-812, respectively. (**B** and **C**) Dual-colour FISH refinement of two target regions. Dual-colour FISH experiment carried out on metaphase chromosomes from IPC-BC-116 (**B**) with digoxigenin-labelled DNA of RP11-164A22 (revealed in red, TRITC) in combination with biotinylated DNA of RP11-99F3 and centromeric probe specific for the chromosome 6 (revealed in green, FITC). The telomeric position of RP11-164A22 allowed the identification of normal chromosome 1 and derivative chromosome 6. The concomitant presence of RP11-99F3 on both derivatives and on chromosome 1 suggested that this clone spans the 1p36 breakpoint. Similarly, dual-colour FISH experiment carried out on metaphase chromosomes from UACC-812 (**C**) using biotinylated DNA of RP11-190J8 BAC (revealed in green, FITC) and digoxigenin-labelled centromeric probe specific for chromosome 12 (revealed in red, TRITC) shows that RP11-190J8 spans the 5p12 breakpoint.

All breakpoints were located, and their positions are shown in Figure 2A. After considering all derivatives for each cell line, a total of 136 breakpoints were described. They were distributed as follows: 47, 29, 28, 18 and 14 on chromosome 1, 5, 8, 12 and 17, respectively. They were located on 4 (chromosome 17) to 11 (chromosome 1) regions per chromosome (Figure 2A) (Supplementary Table 1 and Figure 2A). The same region of breakpoint could be found in one (e.g. 12q13) to seven (1q21) cell lines. The same chromosome was affected in eight to 11 cell lines (Table 1). The regions with more than three breakpoints are referenced in Table 2. For each of them, a breakpoint incidence (BI) was defined for each chromosome as the ratio of the number of events found in this region and the total number of breakpoints observed along the given chromosome (47, 29, 28, 18 and 14 for chromosome 1, 5, 8, 12 and 17, respectively). For each chromosome, a recurrence index (RI) was defined as the ratio of the number of affected cell lines and the total number of tested cell lines. The product BI × RI allowed to point to breakpoints presenting both high breakpoint incidence and high recurrence (Table 2).

### Definition of altered regions on each chromosome

There were 11 altered regions on chromosome 1. The pericentromeric region (p12–p22.1 to q11–q21.2) was the site of the most frequent alterations, involving derivatives in about 80% of the analysed cell lines. Mbanding-FISH also allowed the delineation of deletions on both arms of chromosome 1, while amplifications by duplication or triplication were found only on the q arm.

We defined six altered regions on chromosome 5. The most frequent were at 5p11–p13.2 and 5q23.2–q31.1, and affected 70% of the tested cell lines. These regions were located centromeric to FRA5E (5p14) and close to or within FRA5C, respectively; breaks may thus be the consequence of a particular fragility of these regions. Four frequently altered regions were found on chromosome 8: 8p11–p12, 8q10–q21.1, 8q22.1–q23 and 8q24.1-qter. We identified two frequently altered regions on chromosome 12: 12p11–p12 and 12q24.1-qter. Finally, we identified only one frequently altered region on chromosome 17, at 17q21.3–q22.

### Breakpoint characterisation

To confirm that mbanding-FISH is a useful means of screening for chromosomal breaks, we selected two of the potential breakpoints, one in the low incidence range and the second in the high incidence range. The 1p36 and 5p12 breakpoints of the t(1;6)(p36;p21) and t(5;12)(p12;p11) present in IPC-BC-116 and UACC-812 cell lines, respectively, were studied by dual-colour FISH (Figures 2B–C, respectively). Figure 2B shows the results of a dual-colour FISH experiment carried out on metaphase chromosomes from IPC-BC-116 with digoxigenin-labelled DNA of RP11-164A22 (revealed in red, TRITC) in combination with biotinylated DNA of RP11-99F3 and centromeric probe specific for chromosome 6 (revealed in green, FITC). The telomeric position of RP11-164A22 allowed the identification of normal chromosome 1 and derivative chromosome 6. The concomitant presence of RP11-99F3 on both derivatives and on chromosome 1 suggested that this clone spans the 1p36 breakpoint. The RP11-99F3 BAC clone (AC020587) contains the *MGC15730* gene (hypothetical protein MGC15730) covering 270 kb. This gene has 10 exons and produces two variant transcripts by alternative splicing (UCSC Genome Browser on Human May 2004 Assembly is based on NCBI Build 35 (National Center for Biotechnology Information, US National Library of Medicine 8600 Rockville Pike, Bethesda, MD, USA)). The biological function of the corresponding putative proteins is not known.

The t(5;12)(p13;p11) translocation present in UACC-812 targets the most frequently altered region of chromosome 5 (Figure 2A). The FISH analysis using biotinylated DNA of RP11-190J8 BAC clone (AC021600) in combination with the digoxigenin-labelled centromeric probe for chromosome 12 allowed the characterisation of the breakpoint (Figure 2C). The RP11-190J8 BAC clone contains the 5′ part of the *LOC345557* gene (XM_293875.4) (similar to *RIKEN cDNA B130016O10* gene) expressed as two uncharacterised sequences BX648329.1 (7601 bp) and AK127142 (2432 bp). Currently, the biological function of the corresponding putative proteins is not known. Using the SMART software (http://smart.embl-heidelberg.d e/smart/show_motifs.pl), a phospholipase C catalytic domain (noted ‘PLCXc’) was detected in the XP_293875 protein sequence associated to BX648329.1 mRNA. A link was also found with AK127142, which is probably generated by alternative splicing.

## DISCUSSION

### Multicolour-banding approach: towards a better definition of chromosomal rearrangements in breast cancers

The progress of FISH technologies based on chromosome painting, SKY and multiplex-FISH or multicolour-FISH (M-FISH) (Schröck *et al*, 1996; Speicher *et al*, 1996) has overcome the limitations of conventional cytogenetic methods in the characterisation of complex chromosome alterations observed in cancers. It offers a simultaneous visualisation of all human chromosomes in different pseudocolours, and allows a better characterisation of the rearrangements. However, the definition of chromosomal abnormalities such as intrachromosomal changes or abnormal chromosomal segments could not be approached with these techniques. To overcome these limitations, different multicolour-banding techniques have been developed such as: (i) mbanding-FISH (also called multicolour banding) (Chudoba *et al*, 1999; Liehr *et al*, 2002a, 2002b), which is a modification of M-FISH, (ii) a chromosome bar code technique (Müller *et al*, 1997), (iii) a crossspecies colour-banding technique called Rx-FISH (Müller *et al*, 1998) and (iv) a spectral colour banding known as SCAN (Kakazu *et al*, 2003). These approaches have different levels of banding resolution (Kakazu *et al*, 2003). Very recently, the resolution of discernable bars was increased to 100 bars per haploid chromosome set by including human chromosome-specific probes and more well-defined subregional probes (Müller *et al*, 2004). The analysis of chromosomal alterations using these techniques could reveal new aberrations not visible by other methods, and consequently facilitate the molecular characterisation of target genes.

### Comparison of our results with literature data

Our findings correlate well with what is known for chromosome 1. This chromosome is one of the most frequently affected in breast cancers (Dutrillaux *et al*, 1990; Bièche *et al*, 1993; Pandis *et al*, 1995; Teixeira *et al*, 2002; http://cgap.nci.nih.gov/Chromo somes/RecurrentAberrations). Analyses of breast tumours have established a frequency of breaks as follows: 1p36 (6.5%), 1p22 (6.1%), 1p13 (5.9%), 1q10 (24%), 1q11–12 (7%), 1q21 (9.1%), 1q25 (5%) and 1q42 (5.9%) (Teixeira *et al*, 2002). Frequent allelic imbalances have been found at 1p36 (11%), 1q42–44 (13–14%) and amplification is commonly observed at 1q21. The presence of at least four potential tumour suppressor genes (TSG), at 1p13, 1p22, 1p31 and 1p34-pter, has been suggested (see for a review, Bièche *et al*, 1999). Unbalanced translocations affecting chromosome 1 frequently lead to segmental losses, which could target TSG, while oncogenes could be activated by intrachromosomal amplification (essentially on 1q) or by promoting gene fusion.

As opposed to chromosome 1, aberrations of chromosome 5 are relatively rare in breast cancers (http://cgap.nci.nih.gov/Chromo somes/RecurrentAberrations); they target preferentially the 5p15 region (Teixeira *et al*, 2002). Allelic imbalances have been reported with gains of 5p in 39% of breast tumour cell lines (Forozan *et al*, 2000). Losses of 5q (86%) are common in BRCA1-mutated tumours (Tirkkonen *et al*, 1997).

Chromosome 8 aberrations are very frequent in breast tumours, resulting in losses of 8p and gains of 8q (Mertens *et al*, 1995; Adeyinka *et al*, 2000; Davidson *et al*, 2000; Forozan *et al*, 2000; Kytölä *et al*, 2000; Höglund *et al*, 2001; Rummukainen *et al*, 2001; Struski *et al*, 2002; Teixeira *et al*, 2002; Ferti *et al*, 2004; Online CGH Tumour Database; http://cgap.nci.nih.gov/Chromo somes/RecurrentAberrations). The 8p11–p21 region contains several potential TSG and oncogenes (Adélaïde *et al*, 1998, 2003; Ugolini *et al*, 1999; Conte *et al*, 2002; Ray *et al*, 2004).

We have recently shown by FISH analysis that *NRG1* (8p21) is frequently targeted by recurrent breakpoints in breast tumour cell lines including ZR-75-1 (Adélaïde *et al*, 2003), and in 6% of primary tumours (Huang *et al*, 2004). Moreover, studies by Courtay-Cahen *et al* (2000), as well as our work (Adélaïde *et al*, 2003, and data not shown), have suggested the frequent presence of breakpoints located centromeric to *NRG1* (Gelsi-Boyer *et al*, in preparation). Thus, all breakpoint found in this region using mbanding-FISH were confirmed by FISH using BAC clones showing again that mbanding-FISH is reliable to map breakpoints.

Multiplication of the entire 8q arm and regional amplifications on 8q are common features in breast cancer (Kallioniemi *et al*, 1994; Mark *et al*, 1997). The majority of 8q amplicons encompass 8q24, and comprise the *MYC* oncogene (8q24.12) (Yokota *et al*, 1999; Forozan *et al*, 2000). However, several studies have shown the presence of other amplicons at 8q21, 8q22–23 and 8q24 (Kallioniemi *et al*, 1994; Muleris *et al*, 1994; Fejzo *et al*, 1998, Nupponen *et al*, 1999; Forozan *et al*, 2000; Seute *et al*, 2001), possibly associated with the presence of several common fragile sites (Hellman *et al*, 2002).

Reciprocal translocations t(8;12)(q24;p12), t(12;14)(p11;q11) and t(12;21)(p12;q22) have been reported in breast diseases (Rohen *et al*, 1993; see for reviews Petersson *et al*, 1997; Cavalli *et al*, 2001; http://cgap.nci.nih.gov/Chromo somes/RecurrentAberrations). The 12p13, 12q12 and 12q24 regions have the highest frequencies of breaks (Teixeira *et al*, 2002). In SK-BR-3, the derivative der(12)t(3;12) has been previously identified by SKY FISH (Kytölä *et al*, 2000). The use of mbanding-FISH contributed to facilitate the detection of the inversion and finally to characterise this derivative as del(12)(q24.2)inv(12)(p11q24.2)t(3;12)(?;p11).

Chromosome 17 is often the target of amplification at q12–21 and q23 (Forozan *et al*, 2000; Seute *et al*, 2001), and of unbalanced translocations (Kytölä *et al*, 2000). Among the characterised chromosomal rearrangements, the *BCAS4–BCAS3* fusion transcript has been detected only in MCF-7 cells (Bärlund *et al*, 2002). In our study, a dup(17)(q25q21.3) was detected in UACC-812.

In conclusion, mbanding-FISH is a useful approach to localise recurrent chromosome alterations throughout the genome. However, the relevance of these alterations to oncogenesis is not unambiguous. Some breakpoints may be associated to genome instability and be part of a background of alterations that do not involve cancer genes. In contrast, some other breakpoints may be associated to *bona fide* cancer genes. They may or may not involve fragile sites (Huebner and Croce, 2001; Richards, 2001; Dhillon *et al*, 2003). It is thus likely that we have identified both relevant and irrelevant alterations. In the case of breaks that occur at 8p12, we know that some do target potential cancer genes and are found in tumour samples (Huang *et al*, 2004). The two cases of break we studied in greater detail targeted genes with unknown function. More work is required to determine whether these alterations are involved in oncogenesis. The identification of regions of breaks may provide a repertoire of alterations whose study may reveal interesting candidates.

## Figures and Tables

**Table 1:** Breast tumour cell lines analysed by mbanding-FISH

**Cell lines**	**Chr1**	**Chr5**	**Chr8**	**Chr12**	**Chr17**
BrCa-MZ-01			X		
BT-474				X	
CAMA-1		X	X	X	X
HCC1937	X	X			X
IPC-BC-116	X				
MCF-7	X	X			
MCF-10F	X		X		
MDA-MB-157		X	X	X	
MDA-MB-175					X
MDA-MB-231	X		X		X
MDA-MB-361				X	
MDA-MB-453					X
MDA-MB-468	X		X		
SK-BR-3	X	X		X	
SUM-44		X	X		
SUM-52	X	X			
SUM-149		X	X		X
SUM-185	X	X			
UACC-812	X	X		X	X
ZR-75-1	X		X	X	X
					
Total	11	10	9	7	8

**Table 2:** Regional localisation of the most frequent breakpoints (>3)

**Regional locations**	**Number of breakpoints**	**BI**	**RI**	**BI × RI (× 10^−2^)**
1p22.1–p12	7	7/47	6/11	8.12
1q11–q21.2	11	11/47	7/11	14.9
1q21.3–q24	6	6/47	4/11	4.64
1q25–q31	5	5/47	4/11	3.87
1q32.1–q42.2	5	5/47	4/11	3.87
5p13.2–p10	11	11/29	7/10	26.55
5q22–q31.3	9	9/29	7/10	21.72
8p12–p11	8	8/28	5/9	15.87
8q10–q21.1	4	4/28	4/9	6.35
8q22.1–q23	6	6/28	4/9	9.52
8q24.1–qter	6	6/28	4/9	9.52
12p12–p11	6	6/18	5/7	23.81
12q24.1–qter	5	5/18	3/7	11.9
17q21.3–q22	5	5/14	5/8	22.32

BI=breakpoint incidence; RI=recurrence index.
